# Identity Work in Athletes: A Systematic Review of the Literature

**DOI:** 10.3390/sports11100203

**Published:** 2023-10-17

**Authors:** Yoonki Chun, Elodie Wendling, Michael Sagas

**Affiliations:** 1 Department of Sport Management, University of Florida, Gainesville, FL 32611, USA; 2 Department of Educational Leadership and Sport Management, Washington State University, Pullman, WA 99163, USA; elodie.wendling@wsu.edu; 3 College of Hospitality, Retail, and Sport Management, University of South Carolina, Columbia, SC 29208, USA; msagas@sc.edu

**Keywords:** athletic identity, identity work, transition, injury, role conflict, self-concept, identity development, athlete development, sport psychology

## Abstract

The identity work process allows athletes to achieve a continuous development, revision, and maintenance of themselves. It provides insight into their self-perceptions and particularly intensifies during critical life events. While this process has been widely acknowledged, scant attention has been given to explicitly identifying the specific activities (i.e., identity work modes) involved in athletic identity work and integrating an overarching framework to inform coherent and continuous identities. Thus, we conducted a systematic review of the athletic identity literature to assess how this perspective is represented. Following the PRISMA guidelines, we reviewed 54 articles and analyzed the overall characteristics, bibliographical networks, and accumulated empirical findings. Through this process, we were able to identify the impact of having a strong athletic identity on key variables within and outside of sport. Based on the findings, we examined how identity work modes are depicted and discussed in the literature. Further discussion on how athletic identity literature can contribute to the broader body of knowledge is outlined.

## 1. Introduction

Understanding how an athletic identity is formed, strengthened, and maintained provides a rich opportunity to determine athletes’ self-perceptions and behavioral reactions in various contexts. While athletes, like all individuals, possess multiple identities in various domains of life [1], many scholars in sport psychology have argued that they hold sport as their core life project. In fact, their set of identities is built around their involvement in sport [2,3]. Athletes seeking to pursue an elite sport career may be required to make early commitments during childhood and adolescence to develop and gain competency over others [4]. Putting all their effort into chasing the dream of a college or professional career, young athletes are often raised with an athletic identity that becomes so dominant that they may be unable to think of themselves as anything else other than their athletic identity [5,6,7]. With the increasing competitiveness of youth sport, athletes who have a dominant athletic identity may have an advantage in reaching their athletic goals through a singular focus [8]. Nonetheless, this salience in the athletic identity domain at an early stage of life that pervades and dominates their other identities in life may create unsettling effects on athletes’ overall identity [9].

The ascension of their athletic identity to a prominent position may prevent athletes from exploring other identity dimensions in life and lead them to neglect other aspects of their life [10,11,12,13,14]. However, when their athletic career ends, inevitable shifts in interests and priorities are to be made to adjust to a life after sport. Given that career-seeking athletes were less inclined to explore other aspects of their life outside of sports, they may not have many alternatives to build upon when structuring a new sense of self, which may lead them to experience existential concerns and challenging adjustments upon the termination of their athletic career [6,12,15,16,17,18].

In addition to the adverse impact that a strong and exclusive athletic identity has on the transition process to life after sport, it also affects athletes at varying stages of their careers. For instance, during the development and early mastery stage [19], incompatible demands of academics with athletics throughout an athlete’s career may enact conflicting roles between being an athlete and being a student [11,20]. When academic obligations interfere with athletic duties, and vice versa, it is thought that athletes will experience role conflict, and the burden of the contradictory expectations of being a student and an athlete may cause athletes to feel stressed [21,22]. The amount of time spent practicing and competing and the mental pressure and physical fatigue associated with athletics may leave athletes with limited time and energy to fulfill academic duties and engage in activities outside of sport [20,23]. These competing demands make it difficult for athletes to develop and establish well-rounded, coherent, and integrated identities during adolescence and emerging adulthood, all of which represent a critical developmental time in the identity formation of an individual as he or she transitions into adulthood [24].

While traditionally, the salience of athletic identity has been quantitatively gauged by “the strength and exclusivity of identification with the athlete role” [25], this salience is not considered static and is instead prone to continuous renegotiations throughout an athlete’s career. Specifically, nearing the discontinuation stage [19], it increases the need for negotiated adaptation of athletic identities [18,26], when athletes prepare for their normative (e.g., graduation, expected conclusion of contract) or non-normative (e.g., injury, sudden discontinuation of contract) exit from sports and enter career transitions [27,28,29]. Through these life events, athletes are likely to experience identity threats, during which they can be triggered to reduce the tension from such threats and resolve conflicts by adjusting their identities. Given that the extant literature on identity asserts that individuals’ self-perceptions are not fixed and straightforward but rather multidimensional and dynamic [30,31], identity scholars in management sought to move beyond focusing on the strength of identification and towards a perspective on understanding *identity work* [32]. As the underlying processes of identity development, identity work is defined as the process in which individuals form, repair, maintain, strengthen, or revise their self-meaning [32]. This process demonstrates the fluidity of identities and suggests that no identities are permanent [33]. Acquiring a set of identities does not mean self-meanings are perpetually achieved, but rather, it is a signal of consequent adjustments through continuous identity work.

In the management literature, Caza, Vough, and Puranik [32] developed a conceptual framework for specific activities that individuals engage in for identity work through the identification of four types of identity work modes (i.e., cognitive, discursive, physical, behavioral). Given that the general goal of identity work is to reduce tension and resolve conflicts as a result of facing identity threats [32], applying concepts of identity work modes in the athletic identity developmental process may provide a conceptual framework that provides theoretical understanding of the specific activities involved in overcoming athlete-specific identity threats at different stages of their career, including but not limited to navigating between conflicting roles (e.g., student and athlete), recovering lost athletic identity following injuries and disabilities, and reforming their identity upon career transitions. Furthermore, it can be regarded that identity work occurs over time to establish coherence and continuity between individuals’ multiple identities as well as their personalities and characters [31,34,35]. Thus, the reconciliation of athletic identity with other conflicting identities, the reconstruction of athletic identity among injured athletes, and the reformation of identity upon retirement from a sport career can all be facilitated through different types of identity work modes. While the disparities and potential conflicts between the multiple roles that athletes possess have been acknowledged, it is essential to apply the identity work perspective to exploring contingencies that can enable or inhibit establishing coherence and continuity between roles.

Among the existing reviews of the athletic identity development of athletes, Edison et al. [34] focused on studies specifically investigating the youth athlete population, while Ronkainen et al. [36] reviewed studies that examined athletic identity through cultural epistemology, including methodologies using inquiry and discourse analyses. Corresponding to the suggestion by Edison, Christino, and Rizzone [34], we sought to address the need to expand the literature review to encompass athletes beyond the youth level and at various career stages (e.g., college, professional, retired). According to the position stand on the career development and transition of athletes led by Stambulova et al. [37], an important recommendation was made to practitioners in sport psychology to encourage athletes to reflect on bridging, through identity work, their past, present, and future narratives. However, scant attention has been placed on explicitly identifying the activities that are involved in athletic identity work and on integrating such processes in an overarching framework that would in turn inform identity coherence and continuity, as advanced by the work of Caza, Vough, and Puranik [32]. Therefore, the purpose of the current review is to: (a) outline and map the overall structure of the literature pertaining to athletic identity work, (b) accumulate empirical findings related to the athletic identities of career-seeking elite athletes, and (c) deductively categorize athletic identity work modes using Caza, Vough, and Puranik’s framework [32].

We first assessed the overall trends in the athletic identity work literature, then we organized our review based on the bibliographic coupling analysis in order to better understand the overall structure of the literature and how (dis-)connected the overarching themes of the research on athletic identity have been thus far. In doing so, we provided a summary of the accumulated empirical quantitative results and used primary findings from the existing literature to demonstrate how identity work modes can be applied in this work. Therefore, through this review, we explored how compatible the athletic identity literature is with the broader body of knowledge surrounding the identity work perspective [32] and how it can contribute to further theorization through the unique challenges associated with athletic identity.

## 2. Methods

### 2.1. Article Selection

We adopted a systematic review approach to identify and select the articles that were included in the current study. Under the Preferred Reporting Items for Systematic reviews and Meta-Analyses (PRISMA) guidelines, an explicit methodological approach was used to collect and synthesize literature findings [38]. Specifically, the process involved: (a) establishing eligibility criteria, (b) identifying information sources, (c) developing a search strategy, (d) selecting studies, and (e) extracting and analyzing data.

### 2.2. Eligibility Criteria

Based on the purpose of the current review, we established the following eligibility criteria. First, the studies must be peer-reviewed empirical studies in English that were published prior to 2022 and are relevant to athletic identity and an identity work perspective. Second, we limited the disciplines in which the studies provided implications to sociology, education, psychology, medicine, kinesiology, management, and their sport-related subdisciplines (e.g., sport management). Through this process, articles falling under academic disciplines that were considered irrelevant to the purpose of this study were excluded from the review (e.g., theology, substance behavior). Lastly, during the abstract and full-text review stage, studies that were included in the search due to the use of keywords (e.g., athletic identity) as supplements were excluded. For instance, studies that discussed athletic identity to provide empirical support to their particular topics were determined to be irrelevant to our review.

### 2.3. Information Sources

The databases used to conduct the search for articles were Web of Science, APA PsychNet, and PubMed. Any duplicate articles from these separate searches were removed. Additionally, hand searching was utilized to identify additional relevant studies [38].

### 2.4. Search Strategy

We utilized the search functions provided by the databases to conduct initial searches. The topic keywords included in the search consisted of mainly two parts: athletic identity and identity work. In addition, as the concepts of identity work may be used implicitly without matching terms, keywords conveying similar meanings were also included in the search. As a result, the following search equation was applied sequentially:

1. “Athletic Identity”
2. Work OR development OR formation OR management OR maintain*
3. 1 AND 2
4. Reference lists of retrieved studies were reviewed for hand searching.

### 2.5. Included Articles

**Figure 1:**
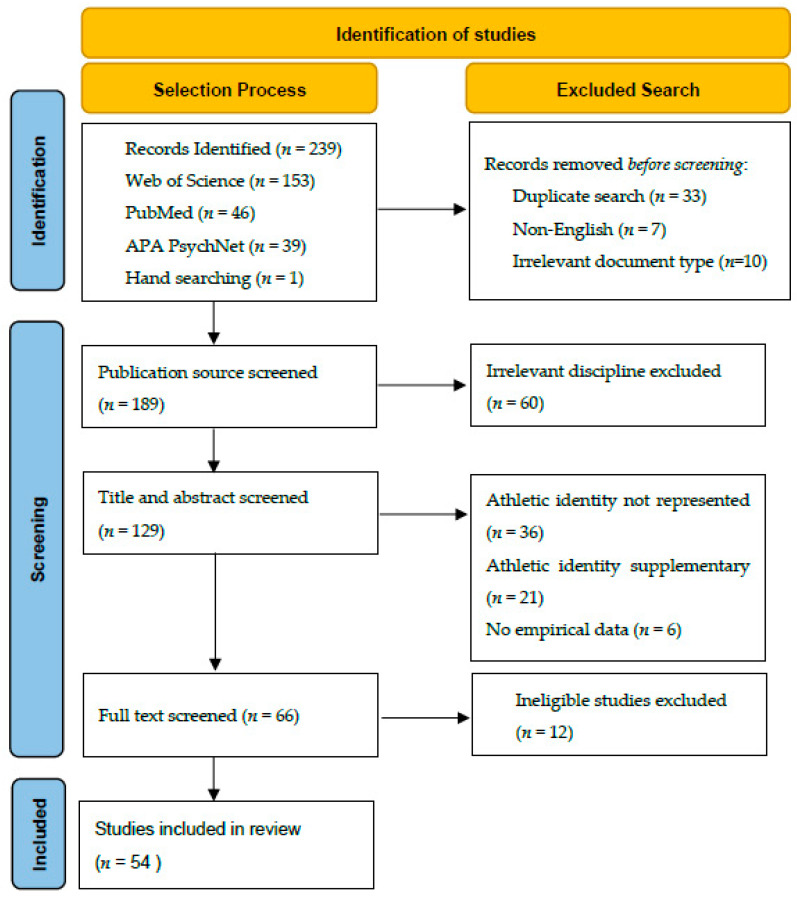
Review article selection flow diagram.

Through the initial search of electronic databases and hand searching, 239 studies were identified. Duplicate studies (*n* = 33), non-English studies (*n* = 7), and irrelevant document types (*n* = 10) were removed (*n*total = 189). Next, publication sources were screened, and studies that provided insufficient implications for our disciplines of interest were removed (*n* = 60, *n*total = 129). For example, we removed journals focusing on topics such as theology, philosophy, or substance behavior (e.g., drug addiction). Furthermore, we analyzed and screened the studies’ titles, abstracts, and keywords based on whether the identity work was central in the article and removed studies (*n* = 63, *n*total = 66) for which identity work was used as an auxiliary concept in the article. Finally, we reviewed the remaining articles by fully following the aforementioned eligibility criteria and ensuring that 50% of the articles were at least double-reviewed, as recommended by Liberati et al. [39]. During this step, additional studies (*n* = 12) were removed if they were identified as ineligible by the reviewers. When a disagreement on the inclusion of the study was raised among the reviewers, a decision on whether to retain the article was made until a consensus was reached. As a result of these steps, we retained 54 studies for the purpose of our review. While alternative approaches in retrieving studies may result in a different set of articles, this systematic process allowed the current review to concentrate on relevant studies and objectively derive meaningful data. In Figure 1, a summary of the systematic flow of the article selection process is provided.

### 2.6. Data Extraction and Analysis

We performed a full-text review from the retrieved articles for data extraction and analysis. First, key study features and the overall trend in the literature were accumulated, including the publication year, research design, sample population, and region. Moreover, the theoretical frameworks applied to the studies were identified, as well as measurement tools, results, and findings. Next, with the complete dataset, we conducted a bibliographic coupling analysis using VOSviewer visualization software, which assessed the strength of the relationship between articles based on the number of references they shared. The software tool constructs and visualizes bibliometric networks through clustering techniques, which is important in identifying groups related to the literature [40]. Through the analysis, we identified thematic clusters within the literature based on how connected or distant the studies were from each other. Two analytical approaches were employed based on the identified clusters from the bibliographic analysis. First, we conducted a sum code classification in which quantitative results provided meaningful insights into assessing the literature, as recommended by Sallis et al. [41]. Through this approach, we were able to examine the spectrum and consistency of the associations between athletic identity and relevant variables and identify statistically significant relationships between variables [42]. Finally, in the subset of articles that addressed the underlying identity work of athletes in-depth, we deductively assessed how identity work modes were to be applied to examine how athletes engage in identity work using the framework of Caza, Vough, and Puranik [32].

## 3. Results

### 3.1. Literature Characteristics

The descriptive features of the articles included in the current review (*n* = 54) are outlined in Table 1. While the publication year of the articles retained in this review ranged from 1995 to 2021, an upward trend in the number of articles related to athletes’ identity work that peaked in 2021 (*n* = 8) was observed. Furthermore, most studies drew from a particular theory or multiple theories related to human identity. Aligning with the dominant approach that athletic identity can be broadly defined as a socially assigned role, some studies (*n* = 10, 18.5%) drew their work from identity (role) theory [43,44] and symbolic interactionism [45], as well as social identity theory (*n* = 3, 5.55%). The studies that focused on the developmental process of athletic identity (*n* = 5, 9.25%) referred to Marcia’s identity status paradigm [46] and Erikson’s identity development theory [35]. Other exploratory studies developed their research questions based on previous findings or drew from various theories, as outlined in Table 1 (*n* = 18, 33.3%).

The majority of the studies were quantitative-based, including 38 studies (70.4%), while 14 studies used a qualitative research design (25.9%), and one study (3.7%) employed experimental and mixed-methods designs. In addition, the study populations that were identified within the dataset included student athletes (*n* = 21), youth athletes (*n* = 9), athletes in general (*n* = 5), elite/professional athletes (*n* = 5), college students (*n* = 4), athletes with disabilities (*n* = 3), athletes with injuries (*n* = 3), former athletes (*n* = 3), and dual-career athletes (*n* = 1). We referred to each study’s main population of interest, not accounting for the samples collected for comparisons or as supplements. The study samples’ mean age, from studies that reported the age of the participants, ranged from 12.5 to 40.1 years, old with a mean age of 23.3 years old and a median age of 21.3 years old. In terms of the regions in which the studies were primarily conducted, most of the studies came from the United States and Canada (*n* = 30), followed by the United Kingdom, Europe (*n* = 17), Asia (*n* = 4), and others (*n* = 3). Finally, for the studies that quantitatively assessed athletic identity using scales or items, most of the studies used the Athletic Identity Measurement Scale (AIMS or AIMS-plus; *n* = 29) devised by Brewer, Van Raalte, and Linder [25], followed by the Academic and Athletic Identity Scale (AAIS, *n* = 3) devised by Yukhymenko-Lescroart [47], the Athletic Identity Questionnaire (AIQ, *n* = 3) devised by Anderson et al. [48], and others (*n* = 5).

**Table 1:** Articles included in this systematic review, listed in chronological order.

Reference	Year	Aim/Focus	Design	Scale	Participants	N
Cornelius [49]	1995	Athletic identity and student developoment	Quant	AIMS	College students	228
Murphy et al. [50]	1996	Athletic identity foreclosure	Quant	AIMS	Student athletes	124
Martin et al. [51]	1997	Scale evaluation (AIMS)	Quant	AIMS	General athletes	78
Brown and Hartley [52]	1998	Athletic identity and career development	Quant	CDI	Student athletes	114
Martens and Cox [53]	2000	Athletic identity and career development	Quant	AIMS	College students	226
Miller and Kerr [29]	2003	Role experimentation, identity work, and student athlete transition	Qual	N/A	Student athletes	8
Hockey [54]	2005	Identity work in the face of prolonged injury	Qual	N/A	Injured distance runners	2
Phoenix et al. [55]	2005	Future aging, body, self, and athletes	Quant	AIMS	College students	179
Lally and Kerr [56]	2005	Career planning, athletic identity, and student role identity	Qual	N/A	College students	8
Jones et al. [57]	2005	Identity work and the role of coaches	Qual	N/A	Retired athlete	1
Killeya-Jones [28]	2005	Identity coherence and role discrepancy	Quant	Identity Collection Instrument	Student athletes	40
Nasco and Webb [58]	2006	Public and private dimensions of athletic identity	Quant	PPAIS	Retired athletes/sport participants	677
Anderson, Masse and Hergenroeder [48]	2007	Scale development (AIQ-adolescent)	Quant	AIQ	Adolescents	2094
Lavallee and Robinson [59]	2007	Identity work and transition	Qual	N/A	Retired gymnasts	5
Anderson and Coleman [60]	2008	Scale development (AIQ-adolescent)	Quant	AIQ	Elementary students	936
Anderson et al. [61]	2009	Athletic identity and physical activity	Quant	AIQ	Elementary and middle school	1339
Anderson [62]	2009	Identity development of adolescent girls with disabilities	Qual	N/A	People with Disabilities	13
Houle et al. [63]	2010	Identity work, age, and sport participation	Quant	AIMS	Student athletes/former athletes/non-athletes	242
Gapin and Petruzzello [64]	2011	Athletic identity and eating and exercise disorders	Quant	Not Specified	Runners	179
Tasiemski and Brewer [65]	2011	Athletic identity and post-injury participation	Quant	AIMS	People with disabilities	1034
Steinfeldt and Steinfeldt [66]	2012	Conformity to maculinity and athletic identity	Quant	AIMS	Student athletes	523
Verkooijen et al. [67]	2012	Athletic identity, burnout, and qulity of life	Quant	AIMS	Elite athletes	123
Carless and Douglas [6]	2013	Processes and consequences of identity development among young elite athletes	Qual	N/A	Elite male athletes	2
Perrier, Smith, Strachan and Latimer [27]	2014	Loss and restorement of athletic identity upon acquiring a physical disability	Qual	N/A	People with disabilities	11
Mitchell et al. [68]	2014	Athletic identity of elite English footballers	Quant	AIMS	Youth footballers	168
Yukhymenko-Lescroart [69]	2014	Academic identity and athletic identity predicting performance and persistence	Quant	AAIS	Student athletes	187
Poux and Fry [70]	2015	Motivational climate and athletic identity foreclosure	Quant	AIMS	Student athletes	101
Reifsteck et al. [71]	2015	Student athletes’ physical activity after college	Quant	AIMS	Student athletes	282
Benson et al. [72]	2015	Goal-discrepant threats and career development	Experimental	AIMS	Student athletes	166
Huang et al. [73]	2016	Athletic identity, college experiences, career self-efficacy, and barriers	Quant	AIMS	Student athletes	345
Sanders and Stevinson [74]	2017	Depressive symptoms among retired professional footballers	Quant	AIMS	Retired male footballers	307
Hickey and Roderick [75]	2017	Prsence and expectation of Possible selves, workplace identities, and athlete transitions	Qual	N/A	Professional football athletes	10
Ryba, Stambulova, Selänne, Aunola and Nurmi [13]	2017	Identity work during significant life events	Qual	N/A	Elite athletes	18
Foster and Huml [11]	2017	Athletic identity and academic major chosen	Quant	AIMS	Student athletes	546
Giannone et al. [76]	2017	Athletic identity and depression	Quant	AIMS	Student athletes	72
Rasquinha and Cardinal [77]	2017	Athletic identity, sport level, and cultural popularity	Quant	AIMS	College students	385
Chang et al. [78]	2018	Athletic identity and athlete burnout	Quant	AIMS	Student athletes	132
Anthony and Swank [79]	2018	Identity development of black college athletes	Quant	AIMS	Student athletes	98
Gustafsson et al. [80]	2018	Athletic identity and burnout	Quant	AIMS	Youth elite athletes	448
van Rens et al. [81]	2019	Career development, dual career athletes	Mixed Methods	AAIS	Student athletes	8/86
Ronkainen et al. [82]	2019	Identity work and role models	Qual	N/A	Adolescent athletes	18
Dean [83]	2019	Identity work and dealing with injury	Qual	N/A	Injury (student athlete)	1
Proios [84]	2020	Prediction of athletic identity	Quant	AIMS	People with disabilities	134
Hagiwara [85]	2020	Scale evaluation (Japanese version of AIMS)	Quant	AIMS	College students	1514
Andrijiw [86]	2020	Identity work and regulation	Qual	N/A	Professional hockey affiliates	16
Graupensperger et al. [87]	2020	Athletic identity, social support, and well-being	Quant	AIMS	Student athletes	135
Yukhymenko-Lescroart [47]	2021	Scale development (AAIS)	Quant	AAIS	College students/high-school students/student athletes	989
Monteiro et al. [88]	2021	Self-efficacy, career goals, and athletic identity	Quant	AIMS-Plus	Elite soccer players	281
Uroh and Adewunmi [89]	2021	Psychological impact of COVID-19 on athletes	Quant	AIMS	Multi-sport athletes	64
Haslam et al. [90]	2021	Social group membership infleunce	Quant	Job Deprivation Scale	Retired athletes	398
Zanin et al. [91]	2021	Identity work and turning points	Qual	N/A	Female youth soccer players	28
Cartigny et al. [92]	2021	Career development and self-efficacy in dual career athletes	Quant	AIMS/AAIS	Dual-career athletes	111
Brewer et al. [93]	2021	Scale development (athletic identity foreclosure)	Quant	SSMIF	Student athletes	712
Boz and Kiremitci [94]	2021	Athletic identity foreclosure	Quant	AIMS	Adolescent athletes/non-athletes	2422

Note: AIMS = athletic identity measurement scale; CDI: college and university form; PPAIS: Public–Private Athletic Identity Scale; AIQ: Athletic Identity Questionnaire; AAIS: Academic and Athletic Identity Scale; SSMIF: Sport-Specific Measure of Identity Foreclosure.

### 3.2. Blibliographic Coupling Analysis

**Figure 2:**
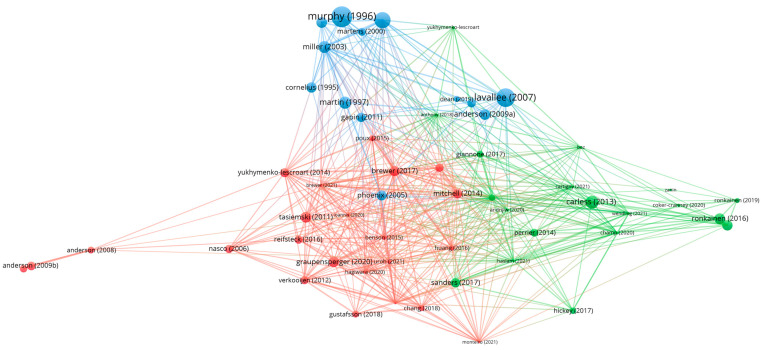
Bibliographic coupling analysis.

A bibliographic coupling analysis was performed to assess the overall structure of the field and how (dis-)connected research on athletic identity has been thus far. As a result, three distinct clusters were identified, as shown in Figure 2. At the bottom right of the figure (cluster 1, *n* = 23), a cluster that is composed of studies that mainly focus on examining the strength of athletic identity among student athletes and adolescents was formed e.g., [61,78,84]. In this cluster, all the studies attempted to measure the samples’ athletic identity quantitatively through widely used instruments such as the AIMS. The predominant theory used for this group was identity (role) theory. Next, at the bottom left corner (cluster 2, *n* = 18) and the top middle (cluster 3, *n* = 13) of the figure, studies that shared numerous similarities in terms of theoretical approaches (i.e., identity theory and narrative theory) and methodology (i.e., qualitative) were identified. These two groups of literature focused on athletes’ identity work during critical life events, such as career transitions, e.g., [86,90,94] and identity conflicts and crises, e.g., [51,59,64]. However, only cluster 3 referred to significant life events related to injuries and athletes facing disabilities, while the sample characteristics of being a professional athlete were only present in cluster 2.

### 3.3. Sum Code Classification Analysis

The studies primarily positioned in cluster 1 provided implications for identity work by assessing various correlates of athletic identity. In the sense that identity work includes the initial formation of identities, identifying the variables contributing to this development helps us understand how athletic identity is strengthened or weakened among athletes. The direction and the need for further identity work can be inferred by looking at the various correlates of athletic identity. Given that understanding the scope of quantitative findings related to athletic identity can provide insights into athletic identity work, we accumulated the quantitative results of the included studies and performed a sum code classification. The significance and direction of the relationships between the variables and athletic identity were analyzed, followed by finding the percentage of the sample groups that supported the associations to provide an accumulated sum code. The statistical reports that were in consensus across all the samples were given a code (e.g., +, −) to indicate the direction of the relationship. Otherwise, question marks were assigned when the results were conflicted, and a 0-mark indicated that the relationships were not statistically significant in the studies. Table 2 provides a summary of the results of the sum code classification. While the n column represents the number of studies that assessed the respective variable’s relationship with athletic identity, the k column refers to the number of sample groups that examined the relationship, as some studies had sub-studies that tested the relationships across multiple groups.

First, the variables that showed a positive (+) relationship with athletic identity included goal perspectives, volitional competency, psychological distress, racial identity, sport participation, meaning in life, perceived control, motivational climate, sport obligations, eating disorders, physical activity, conformity to masculine norms, self-efficacy, fitness, social support, sport level, and the cultural popularity of the sport. Second, GPA, career barriers, college resource utilization, future aging of body and self, sedentary behavior, the rigor of the major chosen, and life management were found to have negative (-) relationships with athletic identity. Third, the variables that showed conflicting (?) results were burnout, gender, career preparedness, and psychological well-being. Lastly, the variables that had no relationship (0) with athletic identity were athletic identity foreclosure, living conditions, and vocational identity. Overall, a strong athletic identity was positively associated with variables related to athletes’ physical health, as shown by a more active lifestyle and an increased participation in sporting activities. Although the self-perception of being an athlete may have intensified the factors that contributed to athletic performance, intense involvement in sport was correlated with athletes experiencing tremendous pressure and psychological distress, leaving them at psychological risks (e.g., depression, anxiety, eating disorders). While inconsistent findings were observed regarding career preparedness, a strong athletic identity may have had a negative impact on academic achievement and overall college experience.

### 3.4. Identity Work upon Significant Life Events

As outlined by Caza, Vough, and Puranik [32], three theoretical assumptions were developed in terms of individuals’ identity work. First, identity work is assumed to be an ongoing process involving experiments and reconstructions of one’s identities [95,96]. Second, significant events such as transitions, identity conflicts, and challenging environments can initiate or intensify identity work [97,98]. Indeed, individuals are more likely to engage in a deeper level of identity work upon encountering critical life events. The articles that were identified in clusters 2 and 3 shared such perspectives. Case of injuries, transitions, role conflicts, and acquiring life-long disabilities may present individuals with identity threats that endanger their identity as athletes, which in turn would require them to engage in further identity work. For instance, Perrier, Smith, Strachan, and Latimer [27] reported how injured athletes utilized their master narratives as athletes (e.g., performance, goal orientation) to engage in identity work that helped reconstruct their lost athletic identity.

In the third assumption, Caza, Vough, and Puranik [32] noted that, despite individuals having a certain degree of agency over their identity work, they are also highly interdependent on social contexts [95,99,100,101]. For instance, Anderson [62] elaborated on how social interactions can affect identity work. She reported that the involvement of girls with disabilities in wheelchair sport programs facilitated their identity development through various socializing agents (e.g., peers, role models, family members). A related finding expands on role models, where athletes engage in identity exploration under the influence of role models that share similarities with themselves [82]. Additional factors that influenced athletes’ identity work included group membership [90], sports culture [6], prolonged injury [54], racial identity [79], retirement [75,86,90], and multiple role experiments [13,29]. Collectively, the literature pointed out the significance of the identity work of athletes while implying that such processes are facilitated and affected by personal explorations, critical life events, and social contexts.

### 3.5. Identity Work Modes

As noted, identity work increases at specific points of athletes’ journeys by using four types of identity work modes. While the management literature detailed the dispersed tactics involved in identity work, ranging from mental activity to performance-based and linguistic actions [32,102,103], we inferred from the existing athletic identity work literature how the different identity work modes may be applied in the various critical life events that athletes can face throughout their athletic career. It is noted that although each mode is distinct in form, identity work can incorporate multiple modes simultaneously, working in tandem. In the subsequent passage, we highlight from the reviewed articles, including the major illustrations of each identity work mode independently.

#### 3.5.1. Cognitive Identity Work Mode

Cognitive identity work involves psychological efforts to understand and process an identity [104]. The cognitive identity work of athletes generally involves the internal construction of self-perceptions as athletes. It is characterized as a self-reflective activity of identity work that involves self-questioning, reflexive sense-making, and self-change [32]. For instance, a study on adolescent athletes with disabilities showed that their participation in sports and their continued experience of winning and being strong competitors triggered cognitive identity work, creating self-perceptions as competent athletes [62]. However, it has also been pointed out that athletes’ engagement in isolated identity work that is only focused on the athletic domain may prevent a well-rounded identity from developing. As explained by Murphy, Petitpas, and Brewer [50], athletes’ exclusive commitment to their athletic identity without sufficient exploration of alternatives might result from identity work that is being affected by external demands, which leads athletes to cognitively isolate themselves into the athletic domain. As athletes reflect on themselves as only being athletes, strengthening their athletic identity without establishing a coherent balance between other domains would create what the literature has commonly operationalized as athletic identity foreclosure. Foreclosing on athletic identity can lead to insufficient career development, enhanced role conflicts, adjustment difficulties during transitions, and a greater chance of using performance-enhancing drugs, e.g., [50,58,105].

Nevertheless, athletes’ cognitive identity work may not necessarily need to be confined to strengthening a single identity, as was the case in a study conducted by Yukhymenko-Lescroart [69]. Although athletic identity was negatively associated with student athletes’ academic performance, student athletes demonstrated higher academic persistence when they showed a harmonious passion for sport. Providing a free and autonomous internalization of sport participation assisted in developing a harmonious passion for sport cognitively [69]. Therefore, it can be assumed that student athletes may integrate their strength from having a salient athletic identity into different identity domains through a more balanced approach to the identity work process. In other words, identity work can be regarded as a process that occurs over time to establish coherence and continuity between individuals’ multiple identities as well as their personalities and characters [31,35].

Collectively, it can be inferred that athletes’ cognitive identity work can be influenced by external factors such that the demands imposed by others may lead athletes to exclusively commit to their sport. Nonetheless, it can also be understood that by engaging in cognitive identity work, athletes can utilize their athletic identity in their other domains of life, given the opportunity to do so. Hickey and Roderick [75] mentioned that athletes could reconstruct their athletic identity upon transitions by envisioning their possible future selves. In doing so, other identity domains become salient, which facilitates their sport career transitions by creating coherence and continuity between identities that are essential for athletes upon their exit from sports.

#### 3.5.2. Discursive Identity Work Mode

Discursive identity work mode involves narratives, stories, and conversations, as discourse is vital to one’s identity work. This mode is also known as “identity talk” e.g., [54], which takes into consideration not just the content of one’s stories but also voice tones, word choice, and expressions [32]. This mode enables us to examine how athletes utilize verbal communications to facilitate their identity development. For instance, young Finnish athletes mapped their personal stories through role models as a way of engaging in discursive identity work [82]. This study revealed a gender difference in whom they admired and mapped their identity. While male athletes aligned their narratives to role models who were highly successful athletes or athletes with exceptional skills, female athletes preferred to reflect on athletes with more proximity that matched their needs. Moreover, today’s athletes admire role models who show competency in multiple identities (e.g., athlete and other careers) and were more preferred in creating their personal narratives, as opposed to totalitarian ideologies that were favored in the past. Such a change in trends implies that athletes are increasingly inclined to develop a more multidimensional and coherent set of identities, moving away from solely focusing on their athletic ones.

Furthermore, injured athletes may engage in discursive identity work to enable the reconstruction of their athletic identity, e.g., [27]. As the acquisition of injuries threatened athletes’ identities, it was reported that they focused on constructing narratives around their sport commitment and their potential to remain competitive as an effort to reconstruct their endangered athletic identity. Additionally, the process was gradual, as shown by stronger athletic identity levels among athletes who had sufficient time to generate appropriate narratives post-injury [27]. In a similar context, narratives and conversations on successful past athletic experiences (glorified past selves) were found to be an effective way of engaging in discursive identity work for injured athletes [54]. Engaging in conversations with teammates who shared their past athletic achievement and reminiscing their past selves through stories assisted in maintaining their damaged athletic identity.

According to Ryba, Stambulova, Selänne, Aunola, and Nurmi [13], dual career athletes have been reported to develop narratives that differed in how they perceived their engagement in multiple roles. Although most dual career athletes formed an interdependent relationship between education and sport, some athletes reported to display a monophonic or dissonant style where they felt a sense of incoherence in enacting multiple roles. Thus, the use of the discursive mode of identity work enables us to identify potential guidance in forming coherent identities between the academic and athletic domains of life. In the case of sport retirement, Giannone, Haney, Kealy, and Ogrodniczuk [76] discussed how encouraging athletes to engage in pre-retirement psychotherapeutic interventions that focus on developing identities outside of sport can help to alleviate the psychological distress athletes experience when they talk about their athletic identity and the impact on their transition experience. Engaging in discursive identity work may help lessen the level of anxiety that highly committed athletes report experiencing months following their retirement from sports.

#### 3.5.3. Physical Identity Work Mode

Athletic identity work utilizing the physical mode primarily involves physical appearances or objects that represent athleticism, such as sporting gears and apparel. In other words, our own body, physical objects, or environments that represent ourselves can be actively used when conducting this particular type of identity work. For instance, Hockey [54] reported that injured runners intentionally used high-end sporting goods, such as racing shoes and Gortex jackets, to match their pre-injury displays. Through this use of these physical objects, lost athletic identities were recovered and maintained. As physical identity work is most visible to the wider public, it can often be used to align others’ impressions with self-meanings without engaging in conversations or specific behaviors [32].

Furthermore, the physical mode of identity work is of importance when athletes retire from their sport careers and experience important body changes. Decreased physical prowess, bodily pain, potential weight gain, and/or muscle loss can negatively affect athletes’ physical self-worth and condition [55,59]. The loss of physical capabilities and muscular strength ensuing after decreased training may add to the difficulties of transitioning once their sport careers are over. In the wake of their changed physical form post-retirement, athletes may feel shame and embarrassment and experience negative perceptions of their body image. This dissatisfaction with their body image can lead athletes to experience disordered eating habits upon retirement, which has been found to be a prominent issue in the transitions of former gymnasts, for instance [59]. Therefore, the loss of control over their physical appearance can be upsetting and distressful for former athletes during the transition process, which can prolong their adjustments to a life after sport [59].

Aside from our assumption that such a display of physical objects reflecting athletes’ identity must be prevalent across all sport levels, it was less recognized as a form of identity work within the literature. To the extent that physical objects are easily observable and comparable across athletes in various contexts, especially among young athletes who are sensitive towards their appearance, it is anticipated that physical identity work may hold potential for scholars to touch upon and incorporate in for future athletic identity studies.

#### 3.5.4. Behavioral Identity Work Mode

Behavioral identity work includes specific actions that individuals perform as part of their identity work [106]. Self-verification is the core attribute of this type of identity work because interpersonal behaviors provide utility for establishing self-meaning to match others’ perceptions of themselves [107]. Athletic identity work involving interpersonal behaviors was observed in several articles within our search. In the case of injured athletes, by continuing their pre-injury training routines, they were able to stay committed to their athletic roles and work on sustaining their athletic identity [54]. In addition to maintaining self-perceptions as athletes, behavioral identity work involves athletes’ effort to be recognized and verified by important others, conforming to their expectations. Because athletes are often predominantly perceived to be fit in terms of physical appearance, Gapin and Petruzzello [64] reported that runners with a high level of athletic identities were prone to displaying disordered eating behaviors for self-verification purposes.

Alternately, athletes can also voluntarily enact certain behaviors to reinforce their identities as athletes, highlighting their athletic selves to others. Conformity to masculine norms, taking risks, engaging in violence, and displaying playboy-like images are ways that athletes express and strengthen their athletic identity through their behaviors [66]. Moreover, athletes’ intense daily practice routines, centered around their roles within sports, can be applied as behavioral identity work that may promote an isolated athletic identity [59]. This skewed adaptation of behaviors can potentially result in an incoherent set of identities in which athletes may find themselves only as athletes, neglecting other important identity domains. In other words, the literature implies that by observing the spectrum of behaviors that are applied to or chosen by athletes, it can also be understood whether they are undergoing modes of identity work that are multidimensional and coherent or isolated within a single identity domain.

## 4. Discussion

Throughout the careers of elite athletes, the sports they participate in are usually placed at the center of their life [108]. Their initiation stage starts early, around the age of 10 [19], and they must spend countless hours training and competing to rise through the subsequent development stages [4,109]. As athletes tend to make premature commitments to their athletic careers during their childhood, their identity development offers a unique context for identity scholars in the application of identity work concepts. Not only would such early commitments often be unusual in the career paths one would pursue during adulthood, but also, the salience of the athletic identity domain at such an early stage of life may create conflicting roles, such as being a student and an athlete simultaneously [11], and may not be conducive for athletes to expand their horizons beyond athletics [9]. Lastly, athletes often face early career transitions, during which the loss of a salient athletic identity was observed as one of the most distress-causing variables upon retirement from their athletic career [6,10,12,110,111,112]. At the termination of their athletic career, athletes may have limited identity options to restructure a new sense of self, causing them to experience a challenging transition as they enter adulthood.

While for elite athletes pursuing careers in sports, athletic identity must be the most prominent among all the identities they possess, it can be asserted that a better understanding of athletes’ self-perception through identity work is vital in facilitating multiple role integration and providing guidance in establishing coherence between the athletic identity and other identity domains of their life. Therefore, in this systematic review, the authors, assessed the athletic identity work literature by providing overall trends and accumulating empirical quantitative findings and the key applications of the identity work modes observed within the selected articles. Recently receiving a closer attention, athletic identity work literature has emerged from multiple streams of conceptual, theoretical, and methodological approaches, with the majority of them employing a quantitative research design, measuring athletic identity using AIMS, and using a sample of college student-athletes from the United States and Canada. The studies drew from a wide range of theories such as identity (role) theory [43], narrative identity theory [113,114], social identity theory [115], and Erikson’s (33) identity development theory.

While our review identified a wide range of identity-related theories, based on our bibliographic coupling analysis, literature on athletic identity work is conceptually and empirically clustered into three distinctive groups. Cluster 1 predominantly implemented a quantitative methodological approach, assessing relationships between athletic identity and other key variables within and outside of sport. Applying sum code classification to the accumulated findings, we were able to not only identify the positive associations between a strong athletic identity and physical activity, sport participation, and athletes’ performance, but also recognize the psychological distress that highly committed athletes were reported to experience. In addition, the accumulated findings were aligned with the adverse association of a strong athletic identity and the development of coherent identities and post-career preparations. The negative associations between athletic identity and academic performance and personal activities outside of sports demonstrated the imbalance between athletics and academics while in college. The notion that highly committed athletes may fail to prepare for a life after sport and transition to a new career domain was primarily supported, unless the environment in which the athletes developed was conducive to exploring outside of their sport involvement. In this case, athletic identity did not appear to be related to certain aspects of career preparedness and vocational identity.

Exclusive identity commitment to sports has been a recurring pattern observed in the athletic identity literature, conceptualized as athletic identity foreclosure, to demonstrate how athletes are highly committed to their role in sports without having sufficient time and opportunities to discover alternative identities [116]. Athletes that were exclusively committed to their role in sports were found to be lacking career-related development and displayed difficulties during transitional periods [7,117]. However, previous work has failed to take into consideration the process of identity exploration in determining identity foreclosure, which may explain why there was a lack of a significant relationship between athletic identity and athletic identity foreclosure. Only measuring the strength of athletic identity commitment may only provide a partial explanation of how identity work occurs among athletes and results in certain outcomes.

Relatedly, it is noticed that the segment of studies in cluster 1, in addition to primarily focusing on the strength of athletic identity commitments, seldomly considered the dynamic interaction between the multiple identities athletes may have that uniquely contribute to each of the psychological and behavioral outcomes. Inconsistent findings observed in this group of studies may stem from the fact that most studies failed to account for other identities that athletes possess and how they may be in coherence with athletic identity. Given that identity work research in management has focused on individuals’ possession of multiple roles and group memberships [118,119], it is acknowledged, in this review, that identities are not solely confined by specific attributes (e.g., gender, race, nationality) but integrated through multiple attributes from the past, present, and future [120]. Traditionally, however, the athletic identity literature suffers from a lack of consideration of multiple identities within their studies [119,121]. Thus, a shift in focus towards an integrated athletic identity work perspective using the work modes as a framework is suggested in this study and recommended for future studies.

In contrast with cluster 1, clusters 2 and 3 primarily focused on elaborating on the developmental aspect of athletic identity and the identity work processes involved in this development. By applying relevant theories (e.g., narrative theory, multidimensional model of identity), these two clusters focused on athletes’ subjectivity by using qualitative methods and incorporating multiple identities. The research that focused on identity narratives has been inclined to emphasize that personal stories regarding individuals’ multiple identities must be integrated across domains to establish a coherent self [31]. In the current context, for instance, Hickey and Roderick [75] pointed out that athletes’ self-possessed identities outside immediate athletic domains (e.g., son, brother, friend, student) were also important factors affecting athletic identity work. This assessment would support the assertion that athletes are not theoretically reducible to a unidimensional athletic identity, and that the concept of exclusive athletic identity cannot be the principal conceptualization used to explain athletes’ identity work [75]. It was found that the studies in clusters 2 and 3 were more closely aligned with the overall scope of the identity work perspective. Therefore, in this review, we accumulated important applications that illustrated the range of activities that athletes engage in for identity work using the identity work modes framework.

Given that athletic identity is prone to being continuously renegotiated following diverse life events, it is critical to integrate the identity work perspective within the athletic identity literature to better understand the development, revision, and maintenance of identities among athletes. It can be asserted that acknowledging these activities can further identify the specific moments that athletes conduct intensified identity work and potentially provide adequate support for their further development. In an attempt to resolve the athletic identity threats that are incurred during critical life events, athletes may engage in identity work processes through cognitive, discursive, physical, and behavioral modes for reconciliation or integration of multiple roles. These modes can be used to assist athletes to not only strengthen their athletic identity or recover their lost athletic identity resulting from injuries and disabilities, but also to help them navigate between less compatible roles, reconcile conflicting roles, and reform roles upon athletic retirement. The use of these modes can help in identifying the developmental steps in integrating different roles and forming coherent identities among athletes.

Through the identification of these applications, future research is better informed on how to apply these modes to conceptually frame and expand on the activities involved with athletic identity work. Nevertheless, limitations related to the research design of the current studies in this review, being mostly cross-sectional, preclude us from drawing causal conclusions based on these identity work modes regarding the development of an athletic identity that is integrated and coherent with other identities. Future research is to be encouraged to implement longitudinal designs to determine the efficacy of identity work modes by athletes. To enhance confidence in the causal impact of identity work modes on the developmental trajectory of athletes, experimental research designs are presumably of vital importance. Intervention-based studies, during which various strategies of identity work modes are employed with the potential to promote positive and integrated athletic identity development, would help fill an important gap in the athletic identity work literature.

## 5. Conclusions

Athletic identity research has been receiving increased attention over the last 30 years. While the accumulated body of knowledge has contributed to our understanding of the self-perception of athletes at various levels, we aimed in this review to amalgamate the present ideas within the literature and advocate for incorporating and adapting an integrative identity work perspective on athletic identity. The salience of athletic identity is not static and continues to evolve due to various life events (i.e., injuries, incompatible role demands, transitions) that trigger further identity work. Beyond measuring the strength of athletic identity, it is our hope that scholars will further scrutinize the underlying processes of identity development among athletes. It is within our perspective that the athletic identity work of athletes, as an essential topic of interest, is an ongoing discourse with the potential to not only advance key practical implications to professionals working with athletes, but also to propose further theoretical development given the unique aspects of athletic identity. It is our hope that this review will inspire identity enthusiasts to further investigate and advance athletic identity work research through a multidimensional and dynamic approach in order to foster balanced and holistic development of identities.

## Figures and Tables

**Table 2:** Sum code classification of quantitative studies.

Athletic Identity Correlates	n	k	% k Supporting Associations			
			+	−	0	Sum
Goal perspective	1	1	100			+
Volitional competency	1	1	100			+
Burnout	2	3	66.6	33.3		?
Academic outcomes (GPA)	1	1		100		−
Psychological distress	3	3	100			+
Gender (female)	2	2		50	50	?
Racial identity	1	1	100			+
Career barrier	2	2		100		−
College resource utilization	1	1		100		−
Years of participation	1	1	100			+
Career preparedness	5	5		40	60	?
Athletic identity foreclosure	2	2			100	0
Meaning in life	1	1	100			+
Perceived control	1	1	100			+
Motivational climate	1	1	100			+
Obligation towards participation	1	1	100			+
Eating disorder	1	1	100			+
Physical activity	4	4	100			+
Future aging of body and self	1	1		100		−
Conformity to masculine norms	1	1	100			+
Athletic self-efficacy	1	1	100			+
Fitness	1	1	100			+
Sedentary behavior	1	1		100		−
Social support	1	1	100			+
Psychological well-being	2	2	50		50	?
Rigor of chosen major	1	1		100		−
Sport participation level	2	2	100			+
Cultural popularity of sport	1	1	100			+
Living conditions	1	1			100	0
Sport participation	3	3	100			+
Vocational identity	1	1			100	0
Life management	1	1	100			−

n: number articles; k: number of sample groups.
